# Establishing the behavioural limits for countershaded camouflage

**DOI:** 10.1038/s41598-017-13914-y

**Published:** 2017-10-20

**Authors:** Olivier Penacchio, Julie M. Harris, P. George Lovell

**Affiliations:** 1 School of Psychology and Neuroscience, University of St. Andrews, St. Andrews, Fife KY16 9JP UK; 2 Division of Psychology, Social and Health Sciences, Abertay University, Dundee, DD1 1HG UK

## Abstract

Countershading is a ubiquitous patterning of animals whereby the side that typically faces the highest illumination is darker. When tuned to specific lighting conditions and body orientation with respect to the light field, countershading minimizes the gradient of light the body reflects by counterbalancing shadowing due to illumination, and has therefore classically been thought of as an adaptation for visual camouflage. However, whether and how crypsis degrades when body orientation with respect to the light field is non-optimal has never been studied. We tested the behavioural limits on body orientation for countershading to deliver effective visual camouflage. We asked human participants to detect a countershaded target in a simulated three-dimensional environment. The target was optimally coloured for crypsis in a reference orientation and was displayed at different orientations. Search performance dramatically improved for deviations beyond 15 degrees. Detection time was significantly shorter and accuracy significantly higher than when the target orientation matched the countershading pattern. This work demonstrates the importance of maintaining body orientation appropriate for the displayed camouflage pattern, suggesting a possible selective pressure for animals to orient themselves appropriately to enhance crypsis.

## Introduction

The evolution of animal coloration is affected by several constraints including communication, thermoregulation, protection against ultra-violet radiation and visual camouflage^1–3^. Countershading is a patterning where the parts of the body that usually face the direction of maximum illumination, usually the back, are darker than parts facing in the opposite direction^4,5^. The phenomenon is found in many taxa and contrasting environments, ranging from underwater to open land^6–12^. Countershading can play an important role in thermoregulation by increasing solar thermal inflow^13,14^ (but see^15,16^). Such a pattern is also compatible with the hypothesis of protection against UV radiation^13,17^. Further, a long-standing and influential hypothesis is that countershading enhances visual camouflage by counterbalancing the pattern of illumination on the body and thereby contributing to background matching and to reducing cues on body shape^4,5,10,12,18–20^. We have previously modelled exactly how the shape of the animal, its orientation with respect to the sun, and the weather, could alter the optimal countershading for an animal of a particular shape^21^. In this paper we provide empirical evidence that countershading does act as visual camouflage and we demonstrate how tightly the effectiveness of the camouflage is linked to the animals’ orientation and position relative to the sun. Quite subtle variations in position can dramatically affect conspicuousness.

**Figure 1:**
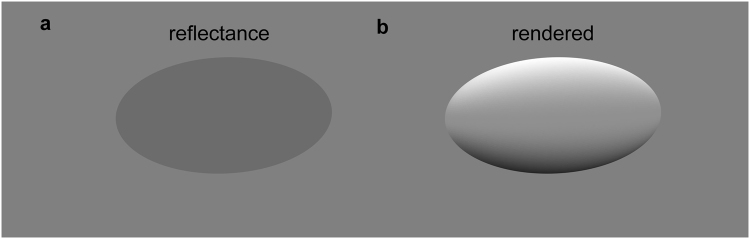
Pattern of irradiance on a uniformly coloured object. (**a**) Uniformly coloured three-dimensional ellipsoid. (**b**) Its appearance when rendered with a sunny sky in the ray-tracing program *Radiance*.

Countershading has been described as contributing to crypsis because it can minimise surface shading that occurs due to self-shadow. Such surface shading can inform judgements of 3D shape^22^. If a uniform object is lit from a particular direction, it reflects more light from the part of its surface facing the light, than the part facing away, as shown in the right panel of Fig. 1. So in sunlight, where light predominantly comes from above, a uniformly patterned animal will appear brighter on top and darker below. If its markings cancel out this shading effect, it has been argued that it could be harder to detect^4,5,10,12,18–20^.

Despite the substantial place countershading has occupied in theories of animal camouflage since the 19^th^ century, just a few attempts have been made to test the effect of countershading on visibility^23–26^. These experiments created artificial caterpillar-shaped baits from dyed pastry and monitored predation rates. The correctly orientated countershaded baits survived longer than control patterns. However, all of these studies have focussed on simple two-tone countershading patterns. These simple experiments were successful in that they proved a point in-principle, but they are not able to get at the subtleties of the effectiveness of countershading. To do that, one requires a general theory and detailed mathematical modelling to predict what specific pattern of countershading is optimal for a particularly shaped animal, in a particular environment. The model we have developed recently^21^ combines the effects of light environment (be it sunny or cloudy weather), angle of the sun (depending on time of day, time of year and latitude), and animal shape to specifically predict what pattern of countershading should be present for any animal and location to minimise the shading on the body.

Using this model we can determine which patterns are theoretically optimal for a given configuration. This allows us to measure in a principled way just how much departure from the optimal countershading profile has an effect on visibility. We used the model to first predict the optimal countershading pattern for a given configuration of a three-dimensional visual scene and then tested the effectiveness of camouflage using visual search.

Our predictions of optimal colour pattern for visual camouflage based on the physics of light allowed us to generate a set of visual stimuli, some with optimal countershading, and some with countershading appropriate for different body orientations in space. Using these, we set up an experiment to measure target visibility, and thus the potential survival value of the target. We used the classic perceptual paradigm of visual search^27–30^. We designed an experiment where human participants search for a target object amidst a series of ‘distractor’ items. Detection speed (measured by reaction time) and search accuracy were recorded as countershading was varied. This approach allowed us to measure whether the countershading pattern has to strictly match the actual properties of a scene to allow enhanced survival and to define a behavioural limit to departure from optimality. A signature of survival will be that, if appropriately camouflaged, targets will be harder to find at all (lower accuracy in participants’ answers), and if found, will take a longer time to find (longer reaction time).

Human participants offer a useful model species for research on camouflage because they are generalist predators with an evolved visual system^31–34^. Humans’ visual processes are well understood, and people can be given complex instructions^31–35^. For example, in the visual search paradigm we measure both accuracy (whether participants choose the correct target) and reaction time (how quickly people detect the target). People can be instructed to be both as accurate and as fast as possible. Using humans allows us to give specific instructions and obtain a much richer dataset.

**Figure 2:**
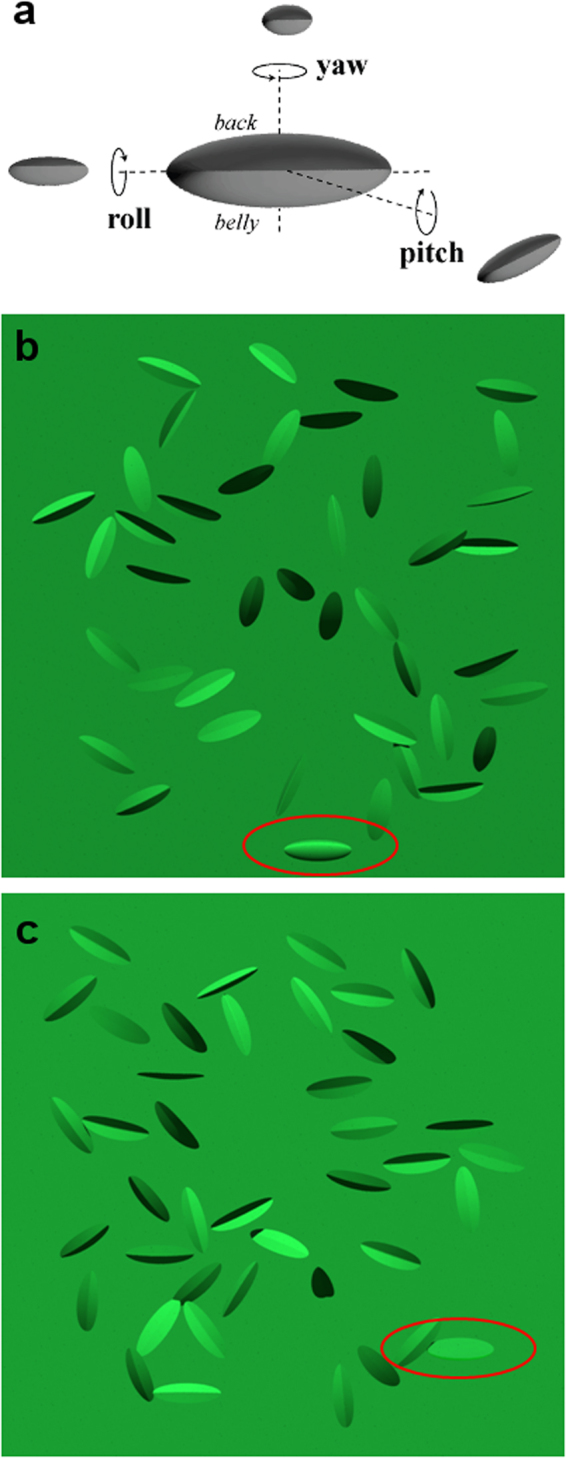
Orientation in space and illustration of Experiment 3: Matching with light distribution. (**a**) Body orientation in space is described by three angles: pitch (−90° to 90°), roll (−180° to 180°) and yaw (−180° to 180°). (**b**) The target object (encircled in red for illustration) is rotated around its long axis (pitch = yaw = 0° and roll = 30°) with respect to the position its countershading profile is optimal for (pitch = yaw = roll = 0°); its shape is obvious from its shading and it pops out in the scene. (**c**) The target object (encircled in red for illustration) has the optimal profile for its actual orientation (pitch = yaw = 0° and roll = 0°); its appearance is therefore flat and it is harder to detect amongst the leaves.

Our stimuli (Fig. 2b,c) are intended to present an analogous problem to that faced by predators of countershaded caterpillars. The animal is roughly ellipsoid while the distractors are similar ellipsoids that have been flattened and folded along their long axis, giving the appearance of leaves. The participants’ task is to detect the ellipsoid target. As the colour and outline of the target and distractors are similar, the only reliable cue to the identity of the target is the variation in shading over its surface. This occurs because the caterpillar has volume, while the leaves are largely flat. It is worth noting that our stimuli do not ever perfectly match the background, their outlines are always visible, just as leaves and caterpillars in the wild would be visible against a background of sky or ground. We wanted our experiment to isolate the effects of countershading on shape perception. This motivation was another incentive for using humans as a model species because they can be instructed to focus on shape in the search task, as will be explained below.

Our computational models predict that for a particular body-position and light environment there is a single optimal pattern of countershading that minimizes shading information on the body^21^. We use visual search experiments, to ask what the behavioural limits on countershading are. For example, if an animal has camouflage optimised for a horizontal stance, how visible does it become if it moves to a 45° pitch? We assessed how target detection was affected by departure of the body orientation of the target object, from the orientation the countershaded pattern on the target object has been optimised for (see Experiments 1, 2 and 3 below). In other words, we seek to specify for the first time just how subtle countershading needs to be to provide effective camouflage that protects an animal from detection.

## Results

### Experiment 1: how departure in pitch affects detection

Participants were shown three-dimensional scenes rendered using the ray tracing software *Radiance*. The scenes contained a target object and several distractors. They were rendered using a single realistic light distribution. The target object was ellipsoidal and its reflectance was optimal for counterbalancing the pattern of irradiance on the body for the light distribution of the scene in the reference orientation (pitch = 0°, roll = 0° and yaw = 0°). The distractors had the form of leaves and were formed as flattened and folded ellipsoids. The target was displayed at five possible pitch values (0°, 15°, 30°, 45° and 90°). Participants were instructed to find a three-dimensional target object, a volume, amongst distractors in form of leaves. We expected camouflage to deteriorate, and thus observers’ detection performance to improve, with departure from the reference orientation.

**Figure 3:**
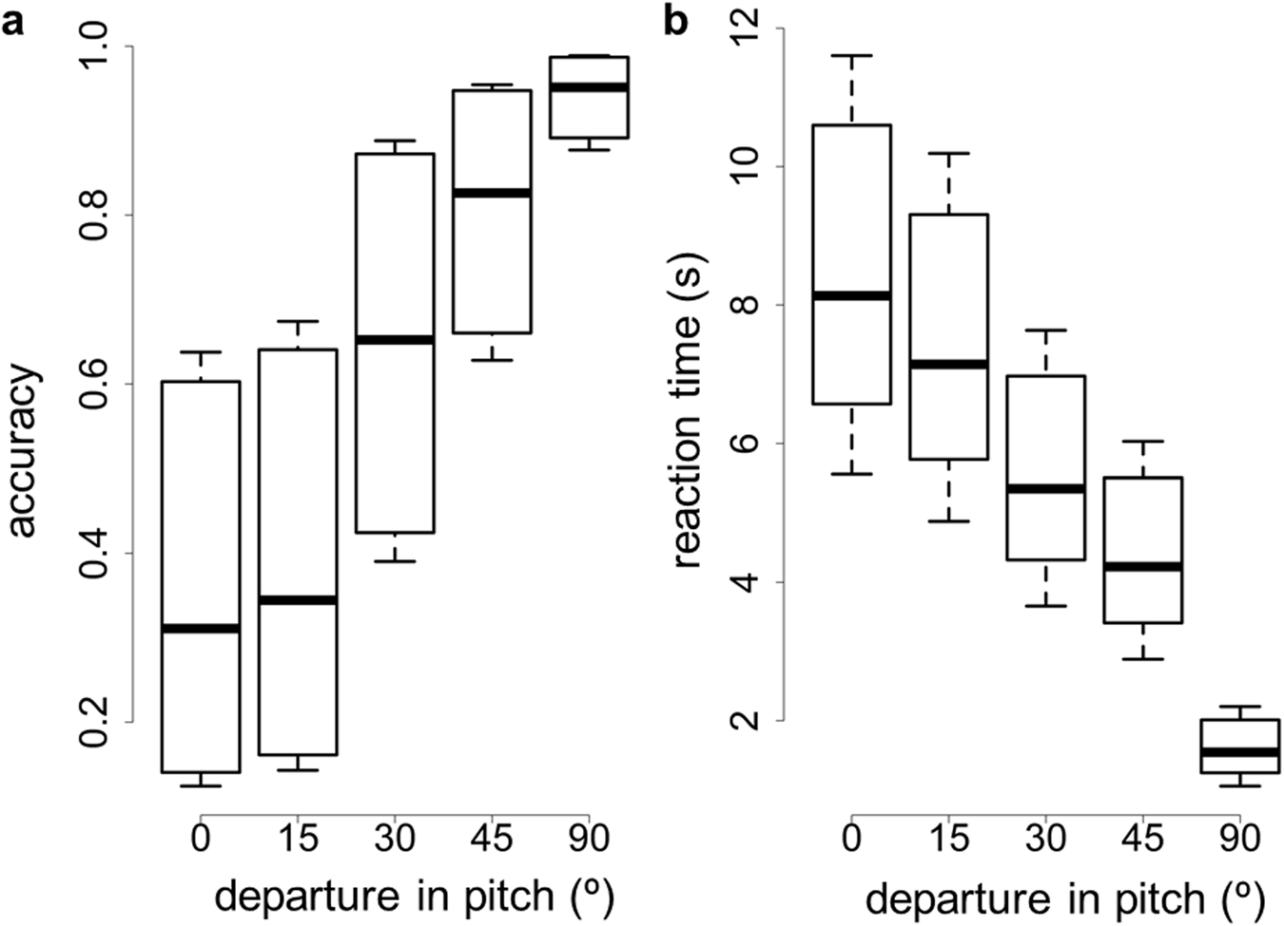
(**a**) Detection accuracy and (**b**) detection speed in function of departure in pitch between the orientation of the target object and the orientation its reflectance was optimally cryptic for (pitch = 0°). Ten observers participated in Experiment 1. Box plots display quartiles, with whiskers extending to the first point within 1.5 inter-quartile ranges of the box.

Figure 3 shows how target detection and reaction time was affected by departures in pitch with respect to the reference orientation (where the target had optimal CS). Target pitch had a strong effect on both detection accuracy (χ^2^ = 285.19, d.f. = 4, p < 10^−15^) and detection speed (χ^2^ = 554.12, d.f. = 4, p < 10^−15^). Detection accuracy fell as the target orientation approached that for optimal camouflage. Multiple comparisons of the different levels of departure are shown in Table 1 (dark grey cells). Similarly, reaction times increased with increasing match to optimal (Table 1, light grey cells). Note that departures from optimality of 15 degrees did not confer a significant reduction in crypsis with respect to the optimum, but that departures of 30 degrees and beyond did.

**Table 1:**
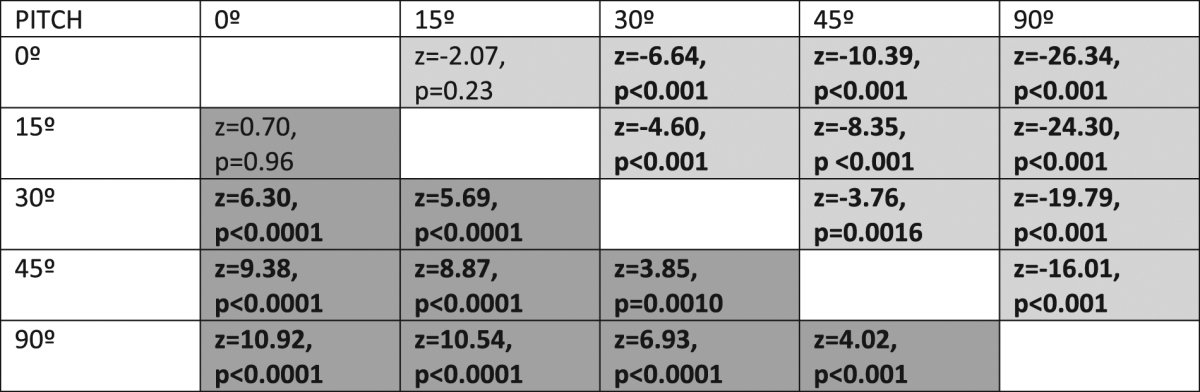
Tukey adjusted test for pair-wise comparisons between orientations (pitch). Entries below the diagonal (dark-grey area) correspond to detection accuracy and entries above the diagonal (light grey area) refer to detection speed. Significant differences below the 0.05 level are shown in bold.

### Experiment 2: how departure in roll affects detection

In Experiment 2 we manipulated the roll of the target instead of its pitch. The target was displayed at five possible roll values (0°, 15°, 30°, 45° and 90°). Observers’ detection performance was expected to improve with departure from the reference orientation.

**Figure 4:**
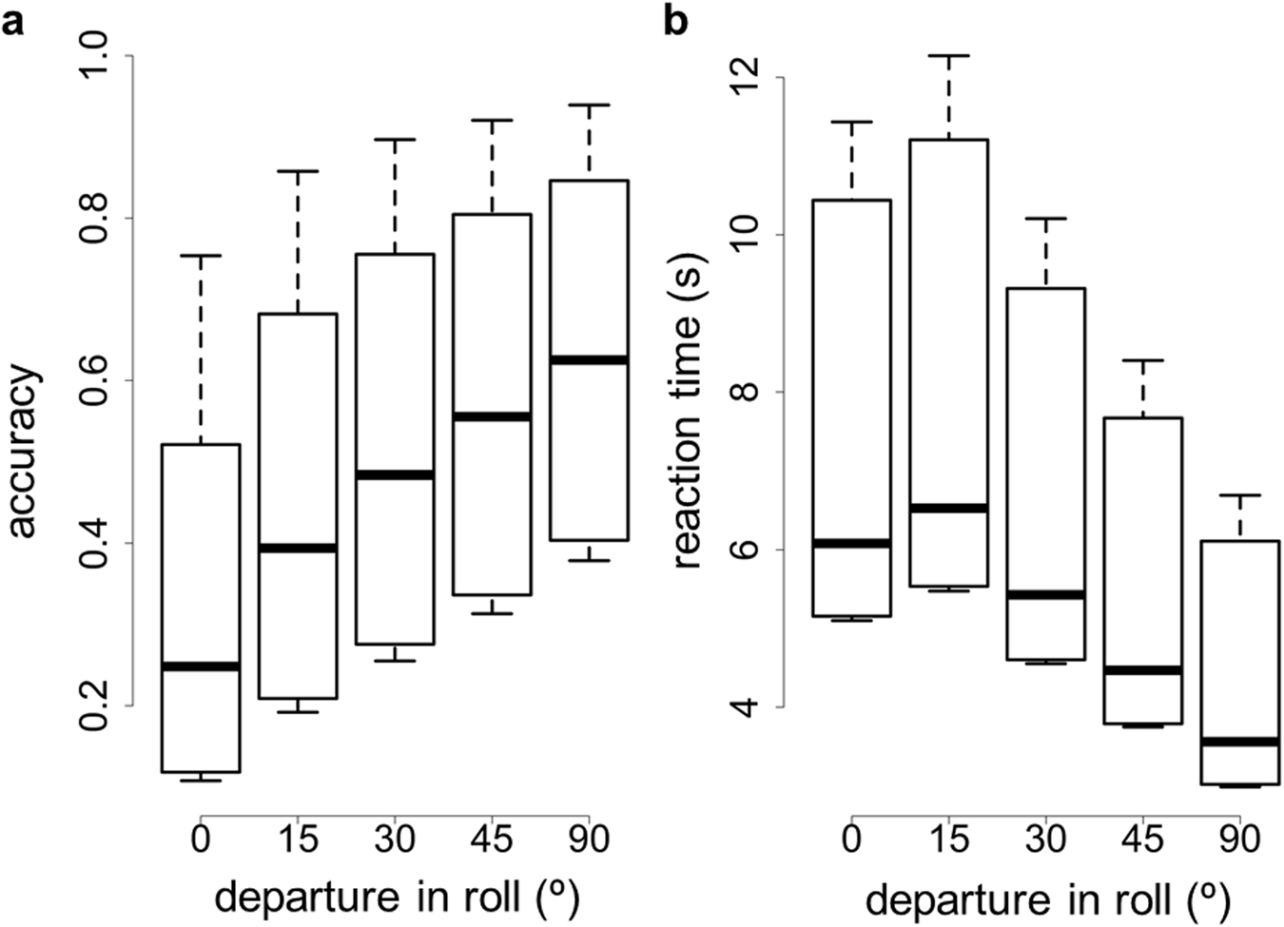
(**a**) Detection accuracy and (**b**) detection speed in function of departure between the roll of the target object and the roll its reflectance was optimally cryptic for (roll = 0°). Seven observers participated in Experiment 2. Box plots display quartiles, with whiskers extending to the first point within 1.5 inter-quartile ranges of the box.

Figure 4 shows how departure in roll from the reference orientation had a strong effect on both detection accuracy (χ^2^ = 40.71, d.f. = 4, p < 10^−7^) and speed (χ^2^ = 60.66, d.f. = 4, p < 10^−11^). Detection accuracy was reduced as the roll of the target approached optimal (Table 2, dark grey cells) and reaction times increased with increasing match to optimal (Table 2, light grey cells). Again, 15–30 degree departure was sufficiently similar to optimal camouflage to fool the visual system almost as well as the optimal camouflage.

**Table 2:**
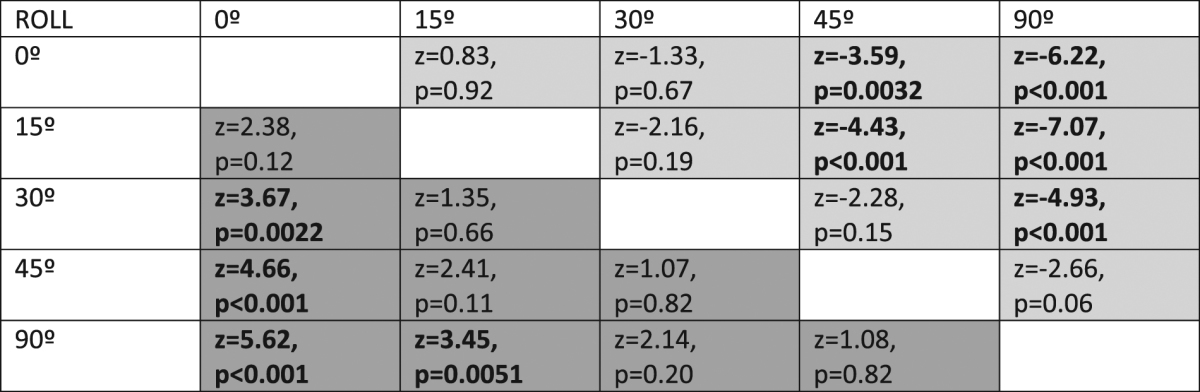
Tukey adjusted test for pair-wise comparisons between orientations (roll). Entries below the diagonal (dark-grey area) correspond to detection accuracy and entries above the diagonal (light grey area) refer to detection speed. Significant differences below the 0.05 level are shown in bold.

### Experiment 3: how departure in yaw affects detection

In Experiment 3 we manipulated the yaw of the target instead of its pitch or roll. The yaw of the target had five possible values (0°, 15°, 30°, 45° and 90°). We anticipated that observers’ detection performance would increase with departure with respect to the reference orientation.

**Figure 5:**
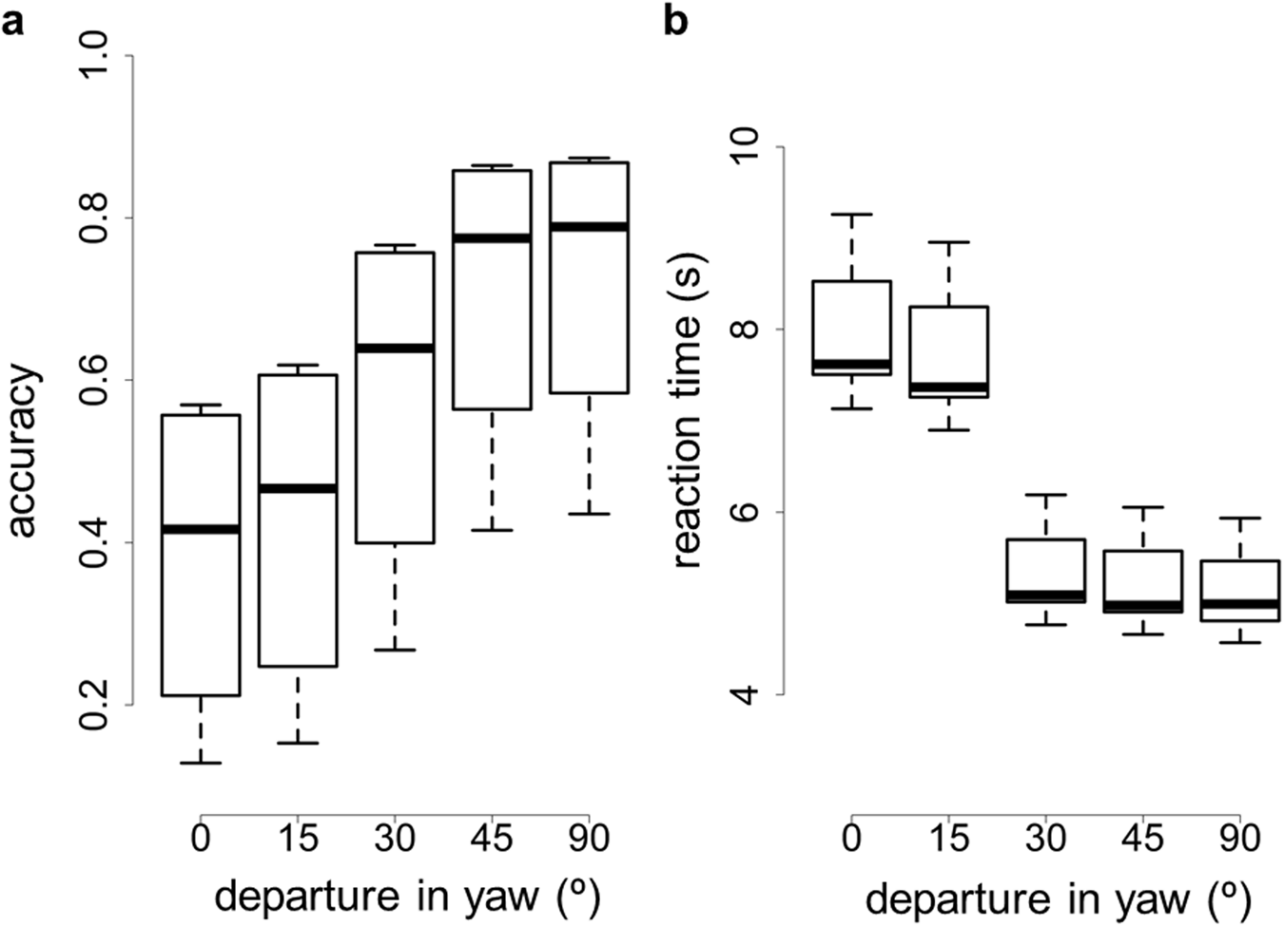
(**a**) Detection accuracy and (**b**) detection speed in function of departure between the yaw of the target object and the yaw its reflectance was optimally cryptic for (yaw = 0°). Ten observers participated in Experiment 3. Box plots display quartiles, with whiskers extending to the first point within 1.5 inter-quartile ranges of the box. Any points beyond the whiskers are plotted as asterisks.

The effects of departure in yaw from the reference orientation are shown in Fig. 5. This manipulation had a strong effect on both detection accuracy (χ^2^ = 63.93, d.f. = 4, p < 10^−12^) and speed (χ^2^ = 50.25, d.f. = 4, p < 10^−9^). In line with the results from Experiment 1 and 2, detection accuracy dropped (Table 3, dark grey cells) and reaction times increased (Table 3, light grey cells) with increasing agreement between target orientation and the orientation its reflectance was optimal for. Again, a departure of 15 degrees from optimal did not deliver results significantly different from optimal.

**Table 3:**
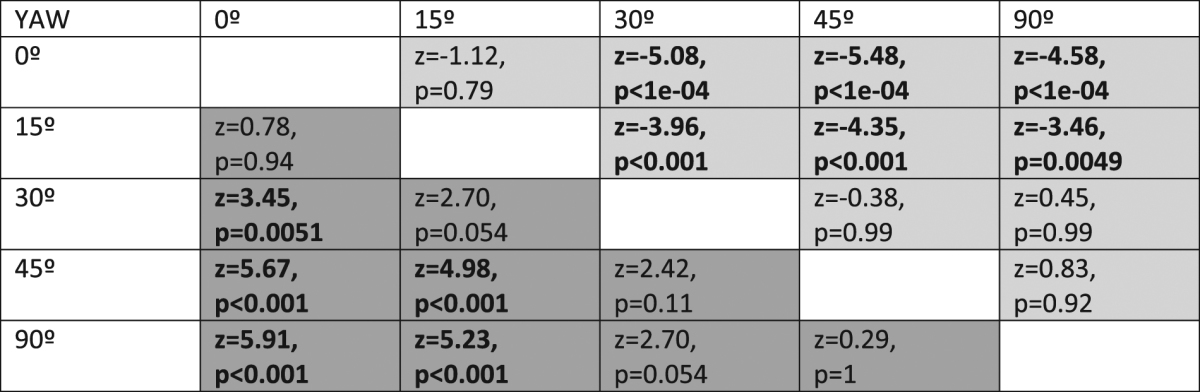
Tukey adjusted test for pair-wise comparisons between orientations (yaw). Entries below the diagonal (dark-grey area) correspond to detection accuracy and entries above the diagonal (light grey area) refer to detection speed. Significant differences below the 0.05 level are shown in bold.

#### Effect of number of distractors on search efficiency

In each experiment we manipulated the number of distractor items (‘leaves’), stimuli either featured 20 or 40 leaves. In visual search tasks an increase in search times as a function of the number of distractors is termed inefficient search^29^. We found that for high deviations (90°) of roll, pitch and yaw from optimal (0°) search is efficient, i.e. there is little additional delay in finding the target when the number of distractor items is doubled (see Supplementary figures S1, S2 and S3 in the Supplementary Material, as well as Tables S1, S2 and S3). Where deviations from optimal are small, the effect of the number of distractors is larger, detection is slower with more distractors, and this suggests that localising the target requires more effort from the participant, possibly that they have to attend to each item in the display before selecting the target.

#### Relation to apparent shading

**Figure 6:**
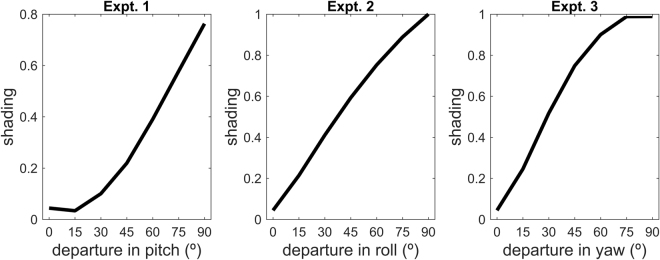
Apparent shading, measured as the difference between the maximum and the minimum of the outgoing radiance, as a function of departure with respect to the reference orientation for (**a**) Experiment 1, pitch, (**b**) Experiment 2, roll, and (**c**) Experiment 3, yaw. The shading has been normalised with the same multiplicative factor across the three experiments such as to have a global maximum of 1.

Experiments 1, 2 and 3 show that visibility increases when the target object orientation departs from the reference orientation. By design, departure in orientation is accompanied by increased shading on the target object, as illustrated by a comparison of the apparent shading in the middle and bottom panels in Fig. 2. To further understand the link between apparent shading on the target body and visibility, we computed the magnitude of the gradient of radiance from the target in each scene. This magnitude is directly determined by the central independent variable in each experiment (i.e., departure with respect to the reference orientation) and does not depend on the other characteristics of the scenes (location of target object relative to distractors, number of distractors). Figure 6 shows how the magnitude of the shading scales with departure in orientation for each experiment. It is not possible to relate functionally the quantities in the three subplots of Fig. 6 to the psychophysically measured accuracies or reaction times in the corresponding experiments (Figs 3–5), since no theory relating the strength of the shading to detection performance is available. However, simple visual comparison of Fig. 6 shows that accuracy and apparent shading exhibit a similar monotonic dependence on departure from the reference orientation. Fig. S4 in the Supplementary material illustrates the monotonic relationship between apparent shading on the target and both search accuracy and detection speed.

## Discussion

In a simulated three-dimensional environment featuring realistic light distributions, ovoid targets were optimally camouflaged by being given reflectance patterns that counteracted countershading caused by the interaction between lighting and body shape. We found that deviations in orientation of greater than 15° in pitch, roll or yaw from the theoretically optimal orientation resulted in statistically significant increases in the speed and accuracy of detection by our human predators. These findings are consistent with the idea that reducing predation by enhancing visual camouflage may be one of the selective pressures on animal colour that led to the countershading reflectance pattern^4,5,10,12,18–20^.

Figure 6 takes the behavioural limits to roll, pitch and yaw identified by the current study and applies these to the results of our simulation study^21^. This is useful because while the simulation results can identify different degrees of optimality for varied body orientations with countershaded camouflage, the simulations do not identify how sub-optimal colouration translates into conspicuousness and possible detection. Of course, any absolute estimate of predation rates must also take into account the nature of the background and the population sizes of both predators and prey.

Notice that deviation in pitch in Experiment 1 has a similar effect as deviation in roll (Experiment 2) and deviation in yaw (Experiment 3). There is, however, a strong difference for pitch in the vertical orientation (90°), for which accuracy was much higher and reaction times much lower than for a deviation of 45°. We think that this could be due to the fact that the outline of the target with a deviation of roll or yaw of 90° was still similar to the outline of several distractors in the scenes, whereas a deviation of 90° in pitch, leading to a perfectly vertical target, was conspicuous as the corresponding orientation was not representative of the general distribution of orientations of the distractors.

In this study we used humans to test the potential function and mechanism of the countershading pattern as camouflage. While a limitation with respect to more ecologically valid predators of caterpillars such as birds, several studies on camouflage have shown strong analogy between field studies with birds as predators and controlled experiments with humans^32,36,37^.

Our experiments have demonstrated the importance of maintaining a body orientation that is close to that of the reference camouflage – i.e. close to the body position relative to illumination direction for which the countershaded pattern was developed. These experiments also set out a behavioural limit to departures in orientation. Departures of around 15° in roll, pitch or yaw have little effect on conspicuousness, however departures of around 30° have a strong impact on “predation”. We did not test this, but it is also likely that combinations of these deviations would result in even greater visibility to our human predators. We can speculate about the implications this has for prey behaviour: the relatively tight constraints upon body orientation should be associated with a rather constrained freedom of movement for feeding during daylight, probably going along with a feeding opportunity cost.

However, the fact that having the “right” countershading profile for optimal crypsis is fundamental for minimal visibility should be counterbalanced by the observation that under all conditions, irrespective of orientation of the body, a countershaded reflectance pattern on the body was systematically and dramatically less visible than a uniform reflectance pattern (Experiment 1: χ^2^ = 282.90, d.f. = 1, p < 10^−15^ for detection accuracy, χ^2^ = 279.14, d.f. = 1, p < 10^−15^ for reaction time; Experiment 2: χ^2^ = 270.33, d.f. = 1, p < 10^−15^ and χ^2^ = 554.42, d.f. = 1, p < 10^−15^, respectively; Experiment 3: χ^2^ = 317.37, d.f. = 1, p < 10^−15^ and χ^2^ = 588.36, d.f. = 1, p < 10^−15^, respectively), showing that suboptimal countershaded reflectance patterns always provided an advantage over no countershading at all.

There is evidence that a number of animal species are sensitive to the shape-from-shading information that directional lighting provides, and therefore that their perception of shape could be deceived by countershading. Humans are very sensitive to shading^38^, which can convey a reliable perception of shape^39,40^. There is also evidence that non-humans primates^41^ and birds^42,43^ can robustly extract shading information to apprehend shape. There has been an ongoing debate about the role of countershading in camouflage^4^. Is it intended to improve background matching or to conceal shape-from-shading cues – obliterative shading? In fact, to achieve crypsis, both factors must be true, assuming a fairly flat and uniform backdrop, you cannot match your background unless you hide your self-shadow (countershading) but countershading without a background match would leave an apparently flat but still potentially conspicuous animal^21^.

An experiment showing that countershading prevents efficient visual search cannot be conclusive regarding which amongst the three main selective pressures on animal colour (thermoregulation, UV protection and visual camouflage), if any, is central to the evolution of the countershading pattern. These selective pressures are essentially compatible, predicting a darker coloration on the parts of the body oriented towards the sun^44^. In a predation experiment with birds in the wild we showed that the countershading pattern boosts visual camouflage, and that optimally if adapted to the lighting of the scene^45^. This result and that of the current study agree with the long-standing hypothesis that visual camouflage may be one of the forces at play in the evolution of the countershading pattern^19,46,47^. Taken together, these findings, and the convergence of the three important selective pressures on animal colour aforementioned, make the ubiquity of the countershading pattern in the animal kingdom more understandable.

In conclusion, the present study highlights the importance of body orientation with respect to the light field for the countershading pattern to provide the most effective camouflage. Furthermore, the requirement for roll, pitch and yaw to fall close to that for the reference camouflage means that animals should ideally re-orient themselves throughout the day as the sun changes position. The next step, then, would be to explore whether countershaded species actively modify their body orientation with respect to the light distribution to lower visibility and decrease predation risk.

## Methods

### Determining optimal countershading for reducing shading cues

Before stimuli could be developed, we needed to model the environmental conditions to be used in the experiments. To determine the optimal countershading patterns to deliver minimal shading information, we followed the modelling previously described in^21^. This work uses three-dimensional (3D) scenes generated with the ray-tracing rendering software *Radiance* ^48^. In *Radiance*, 3D scenes can be defined using objects’ Cartesian coordinates in a tri-mesh format and next rendered using a specific lighting model. *Radiance* has been shown to allow for an accurate rendering of colour^49^ and includes the standard light distributions provided by the International Commission on Illumination^50^, in which latitude, time of the year, time of the day and sky type (*e.g*., sunny or cloudy) can be chosen. In our scenes the sun position was fixed at azimuth 270°, altitude 45°. This resulted in a scene apparently lit from above and behind the observer. This arrangement was chosen in order to avoid shadows cast by the scene items being used as cues towards the target identity – ‘caterpillars’ would cast a larger shadow due to their larger volume. Using our model, the irradiance (quantity of light) *irr*(*x*) falling on an object at location *x* can be computed for any light distribution. Supposing that object reflectance *refl(x)* is Lambertian (i.e., is matte rather than glossy), the reflectance of the object can then be chosen to be the inverse of its pattern of irradiance, so that: $refl(x)=\pi .ra{d}_{hor.leaf}/irr(x)$, where *rad*_*hor.leaf*_ is the radiance of a horizontal patch of a leaf oriented up (towards the zenith). In such a way, when in the reference orientation, the reflectance pattern on the target object delivers a constant radiance that coincides with that of a horizontal surface with the same reflectance as the leaves. This reflectance pattern on the body, which delivers the minimal shading for the light distribution of the scene^21^ and should therefore be minimally visible, is referred to as the ‘optimal’ countershading pattern for the specific orientation in space of the body and the light distribution of the scene.

### Stimuli for visual search

In order to test the utility of countershading as the position of the animal changes relative to the sun, we needed an ‘animal’ target and distractor items, both presented in an environment that can be readily assembled into an image of a 3D search stimulus. The only cue to the target’s identity should be the shading that reveals its unique shape. In order that other cues to detection do not dominate we need to match the outline, colouration and size of the target and the distractors. We rendered a large 3D scene and had observers viewing a much closer computer screen as if viewing a distant scene through a window. The 3D scene (see Fig. 2b,c) was made up of a vertical green backdrop located fronto-parallel to the viewer and positioned eight metres away. There was an identically coloured horizontal ground-plane. The search stimulus was located upon on an implied, fronto-parallel plane half-way between the viewer and the backdrop. The stimulus consisted of a single ellipsoidal target-item (the ‘caterpillar’, 120mm long and 40mm wide in the 3D model). Distractor items were also ellipsoidal and the same size, however these ‘leaves’ (n = 20 or 40) were flattened along one short axes and subsequently curved along their long axes at different angles (mean and standard deviation, 20°). After folding, distractors were manipulated to have the same 2D shape, on the screen, as target objects.

The spatial location of each leaf and the caterpillar was randomised following a uniform distribution; only scenes in which objects were separated by at least 1.3 times the leaf length were kept to avoid spatial overlapping. In all experiments the orientation of each leaf was randomised (from the horizontal, upward facing default pitch = roll = yaw = 0°, see Fig. 2a) by adding offsets to the pitch, roll and yaw values. Roll and yaw were normally distributed random numbers (Matlab, *normrnd*; mean = 0°, σ = 50° for roll, mean = 0°, σ = 10° for yaw) and pitch had a uniform distribution in [−180°,180°]. The operations described above ensured that each ‘leaf’ had a wide range of surface normals, therefore showing a wide range of brightness levels across the leaf surface.

On-screen, the leaves and caterpillars were 1.27° × 0.4° of visual angle (long and projected short axes, respectively). The overall stimulus size was 10.58° × 10.58° of visual angle. The measured on-screen chromaticities of the target and distractors were matched (see Table 4 below). Radiance rendered images have a high dynamic range. In order to present these images on a computer display with a limited dynamic range we rescaled values so that the brightest pixels (99^th^ percentile) fit within the 0–1 range available within the display system. Values that subsequently exceeded 1.0 were clipped to 1.0. This limited the brightest regions on both leaves and caterpillars. We did not anticipate that this would significantly change our measures of visibility as both the leaves and the caterpillars featured bright regions, hence this stimulus property in isolation should not be a useful cue to the presence of a caterpillar. After clipping, the brightness of items varied from 13 to 35 candelas per m^2^.

**Table 4:** Chromaticities of the elements composing the search scenes in the CIE 1931 space.

Item	CIE 1931 x	CIE 1931 y	CIE 1931 Y (luminosity)
Caterpillar	0.14 [0.13–0.15]	0.52 [0.50–0.52]	19.91 [13.42–34.41]
Leaves	0.14 [0.13–0.15]	0.52 [0.50–0.52]	21.33 [14.80–35.14]
Backdrop	0.14 [0.13–0.15]	0.52 [0.50–0.52]	21.56 [14.92–35.15]

### Apparatus

Images were presented bi-ocularly (same image presented to left and right eyes) via a Wheatstone stereoscope. This was done because we anticipated running an additional study examining the contribution of binocular vision to the detection of our camouflaged targets. In the current report all stimuli have zero binocular disparity. The left and right stimuli were presented on a single screen (Mitsubishi Diamondtron 22 inch) controlled by a Cambridge Research Systems Visage (CRS, Rochester, United Kingdom) display system. The total viewing distance was 810mm from eye to display surface. Responses were recorded with a rotary dial (Griffin Powermate; Griffin Technology, Nashville TN, USA). The frame rate for the display was set at 120 frames per second. Observers were seated, in a dimly lit room, at a table facing the stimulus screen with their chin upon a rest.

### Procedure

We used a two-stage response procedure in order to acquire a reaction time measure that is uncontaminated by the time taken to choose from the range of potential responses^51^. Participants were instructed to press down on the rotary dial when they saw the target item. At this point the stimulus was replaced with a scrambled version for one second, scrambled by breaking the image into squares (20 pixels) and rebuilding the image with the squares arranged in a random order. Participants were then presented with a monochromatic version of the stimulus where each item was represented in uniform grey on a black background, with a single item chosen at random highlighted in red. They then had to rotate the dial until they had highlighted the item they believed to be the target, and then press down again to identify the target and end the trial. Each item was highlighted in a sequence determined by its centre-relative location around the clock-face. All the sessions started with a training set composed of 10 stimuli. The experiments had local Ethics Committee approval (UTREC, reference 10769) and followed the guidelines of the University of St Andrews. Participants were all students of the University of St Andrews recruited using the SONA system and were given monetary compensation for each session. They all gave written informed consent to participate. Participants (N = 10, 7 female, N = 7, 5 female, and N = 10, 8 female, for Experiment 1, 2 and 3, respectively, all students of the University of St Andrews) were presented with 200 scenes rendered using the same light distribution. Targets were given one of the 5 possible variations (in pitch, roll and yaw, respectively), with all orientations evenly represented, leading to 40 scenes for each value in each experiment. The number of distractors (20 or 40) was evenly distributed across the conditions.

### Statistical analysis

We analysed how detection accuracy and reaction times varied as a function of departure from optimal camouflage using generalized mixed models. For accuracy, we used a binomial distribution as a model (target found, target missed). We used Gamma distributions to fit reaction time distributions^52^. In all experiments the predictor was the angle (pitch, roll or yaw) that was modified with respect to the reference orientation (5 levels, 0°, 15°, 30°, 45° or 90°). Participants’ identity was included in the models as random effects. To fit the generalized mixed models we used the function *glmer* from the package *lme4* ^53^ in R (R version 3.1.0). Multiple comparisons between predictor levels were performed using the Tukey procedure from the R package *multcomp* ^54^. Data and the R code for analysis are available on the open science framework (https://osf.io/78qw9/).

## Electronic supplementary material

Supplementary material
