# Supplementary material for: Establishing the behavioural limits for countershaded camouflage
Source: Sci Rep. 2017 Oct 20;7:13672. doi: 10.1038/s41598-017-13914-y (PMC5651847; doi:10.1038/s41598-017-13914-y)
Supplement: Supplementary file 1 — Supplementary material [file 41598_2017_13914_MOESM1_ESM.doc]

**Supplementary Materials for:**

**Establishing the behavioural limits for countershaded camouflage**

**Authors**: Olivier Penacchio^1^,*, Julie M. Harris^1^,*, P. George Lovell^2^

^1^School of Psychology and Neuroscience, University of St. Andrews, St. Andrews, Fife KY16 9JP, UK;

^2^Division of Psychology, Social and Health Sciences, Abertay University, Dundee, DD1 1HG, UK

*Correspondence to: [op5@st-andrews.ac.uk](mailto:op5@st-andrews.ac.uk)

**Effect of number of distractors on search efficiency**

We used generalized mixed models (see manuscript, Section “Statistical analysis”) to explore whether there was a significant difference in performance when the stimuli contained 20 or 40 distractors.

**Experiment 1**

| search efficiency measure | deviation in **pitch** | | | | | |
| --- | --- | --- | --- | --- | --- | --- |
|  | all deviations | 0º | 15º | 30º | 45º | 90º |
| accuracy | n.s.  (χ^2^ = 2.67,  p = 0.10) | n.s.  (χ^2^ = 2.13,  p = 0.14) | n.s.  (χ^2^ = 0.77,  p = 0.38) | n.s.  (χ^2^ = 0.69,  p = 0.41) | n.s.  (χ^2^ = 0.03,  p = 0.85) | n.s.  (χ^2^ = 0,  p = 1) |
| reaction time | **χ^2^ = 50.47,**  **p < 10^-11^** | **χ^2^ = 9.53,**  **p = 0.002** | **χ^2^ = 14.46,**  **p < 10^-3^** | **χ^2^ = 5.80,**  **p = 0.016** | **χ^2^ = 19.67,**  **p < 10^-5^** | **χ^2^ = 5.68,**  **p = 0.017** |

Table S1. Effect of the number of distractors (20 versus 40) on search efficiency (detection accuracy, dark grey area, and reaction time, light grey area) for all the scenes considered together and for each of the different levels of deviation in pitch with respect to the reference orientation in Experiment 1. Significant effects below the 0.05 level are shown in bold. The degree of freedom is one for all the tests (d.f. = 1).


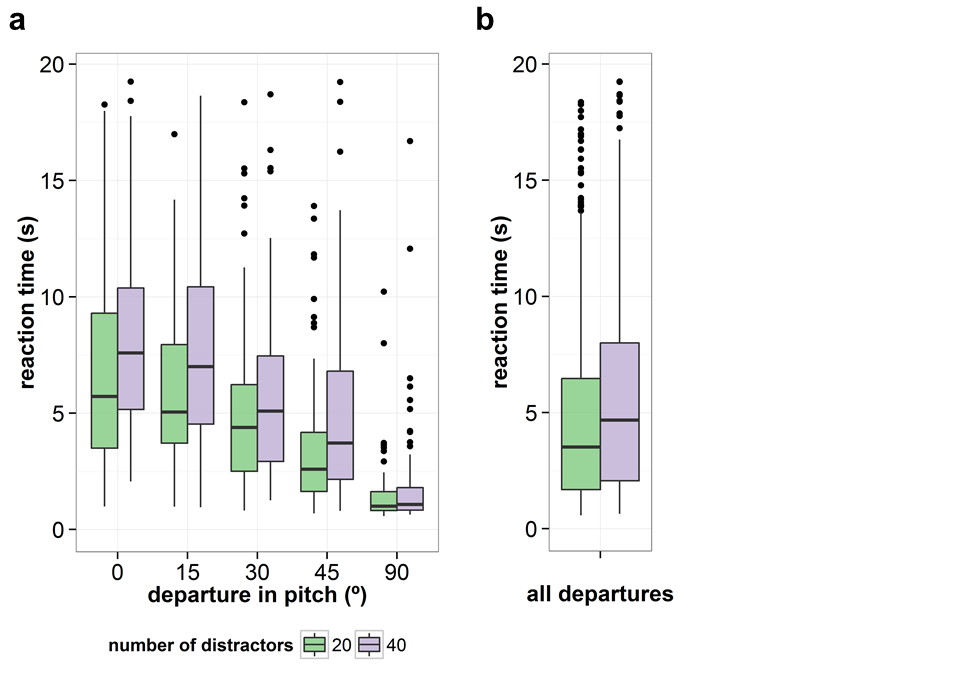


Figure S1. Effect of the number of distractors (20 versus 40) on search efficiency in Experiment 1 for (a) the different levels of departure in pitch with respect to the reference orientation and (b) all the departures in pitch considered together. Box plots display quartiles, with whiskers extending to the first point within 1.5 interquartile ranges of the box. Points beyond the whiskers are plotted as black dots.

**Effect of number of distractors on search efficiency: Experiment 2**

| search efficiency measure | deviation in **roll** | | | | | |
| --- | --- | --- | --- | --- | --- | --- |
|  | all deviations | 0º | 15º | 30º | 45º | 90º |
| accuracy | **χ^2^ = 14.68,**  **p < 10^-3^** | **χ^2^ = 4.18,**  **p = 0.041** | n.s.  (χ^2^ = 2.84,  p = 0.09) | n.s.  (χ^2^ = 1.85,  p = 0.17) | n.s.  (χ^2^ = 1.85,  p = 0.17) | **χ^2^ = 4.35,**  **p = 0.037** |
| reaction time | **χ^2^ = 30.69,**  **p < 10^-7^** | n.s.  (χ^2^ = 3.75,  p = 0.053) | **χ^2^ = 7.41,**  **p = 0.0065** | **χ^2^ = 7.58,**  **p = 0.059** | **χ^2^ = 8.89,**  **p = 0.0029** | **χ^2^ = 5.34,**  **p = 0.021** |

Table S2. Effect of the number of distractors (20 versus 40) on search efficiency (detection accuracy, dark grey area, and reaction time, light grey area) for all the scenes considered together and for each of the different levels of deviation in roll with respect to the reference orientation in Experiment 2. Significant effects below the 0.05 level are shown in bold. The degree of freedom is one for all the tests (d.f. = 1).


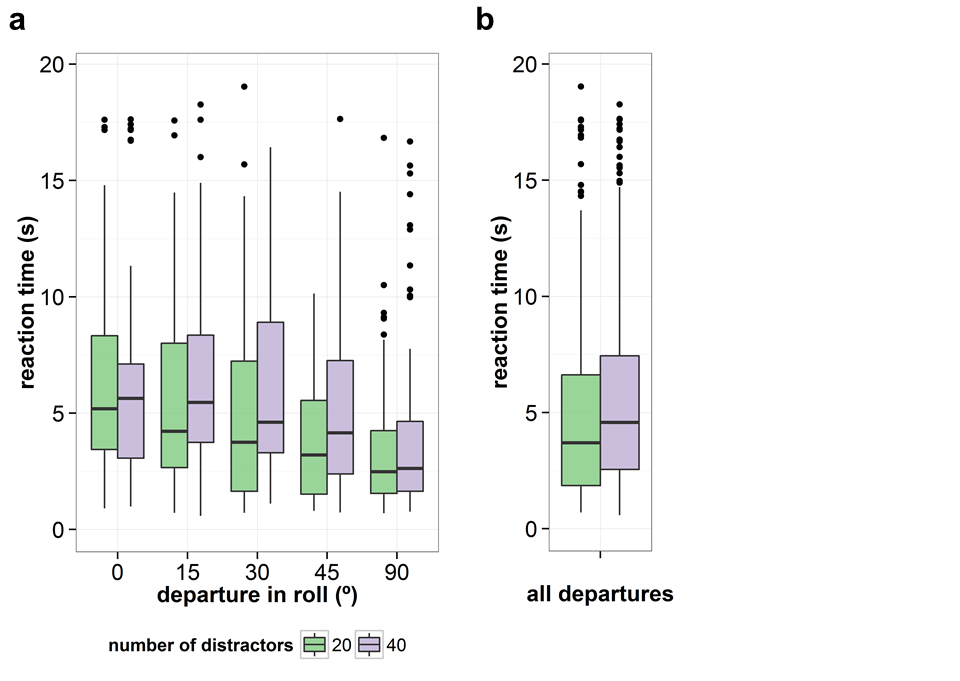


Figure S2. Effect of the number of distractors on search efficiency (20 versus 40) in Experiment 2 for (a) the different levels of departure in roll with respect to the reference orientation and (b) all the departures in roll considered together. Box plots display quartiles, with whiskers extending to the first point within 1.5 interquartile ranges of the box. Points beyond the whiskers are plotted as black dots.

**Effect of number of distractors on search efficiency: Experiment 3**

| search efficiency measure | deviation in **yaw** | | | | | |
| --- | --- | --- | --- | --- | --- | --- |
|  | all deviations | 0º | 15º | 30º | 45º | 90º |
| accuracy | **χ^2^ = 4.22,**  **p = 0.040** | **χ^2^ = 9.22,**  **p = 0.0024** | **χ^2^ = 5.89,**  **p = 0.015** | n.s.  (χ^2^ = 1.67,  p = 0.26) | n.s.  (χ^2^ = 1.45,  p = 0.23) | n.s.  (χ^2^ = 0.16,  p = 0.68) |
| reaction time | **χ^2^ = 27.28,**  **p < 10^-6^** | **χ^2^ = 11.63,**  **p < 10^-3^** | **χ^2^ = 27.80,**  **p < 10^-6^** | n.s.  (χ^2^ = 0.008,  p = 0.93) | **χ^2^ = 8.71,**  **p = 0.0032** | n.s.  (χ^2^ = 0.96,  p = 0.33) |

Table S3. Effect of the number of distractors (20 versus 40) on search efficiency (detection accuracy, dark grey area, and reaction time, light grey area) for all the scenes considered together and for each of the different levels of deviation in yaw with respect to the reference orientation in Experiment 3. Significant effects below the 0.05 level are shown in bold. The degree of freedom is one for all the tests (d.f. = 1).


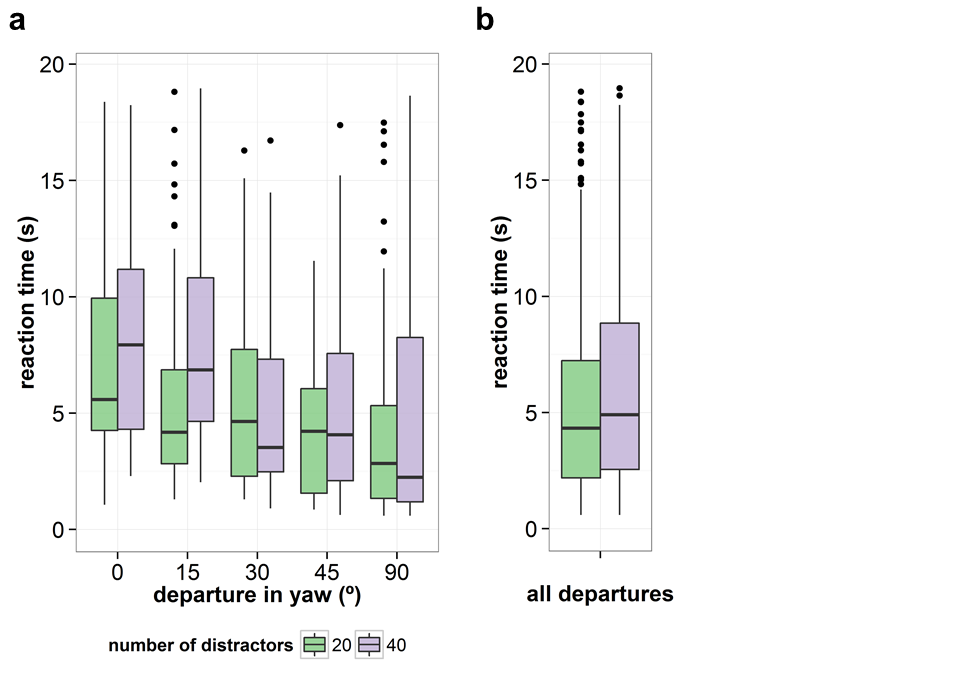


Figure S3. Effect of the number of distractors (20 versus 40) on search efficiency in Experiment 3 for (a) the different levels of departure in yaw with respect to the reference orientation and (b) all the departures in yaw considered together. Box plots display quartiles, with whiskers extending to the first point within 1.5 interquartile ranges of the box. Points beyond the whiskers are plotted as black dots.

**Relationship between search efficiency and apparent shading on the target object**

Apparent shading of the target object is measured as the difference between the maximum and the minimum of the outgoing radiance. Below we illustrate the relationship between observer responses (in terms of accuracy and reaction time) and apparent shading. It is not clear what the mapping should be between response and apparent shading, hence we have not attempted to model a link between the two parameters.


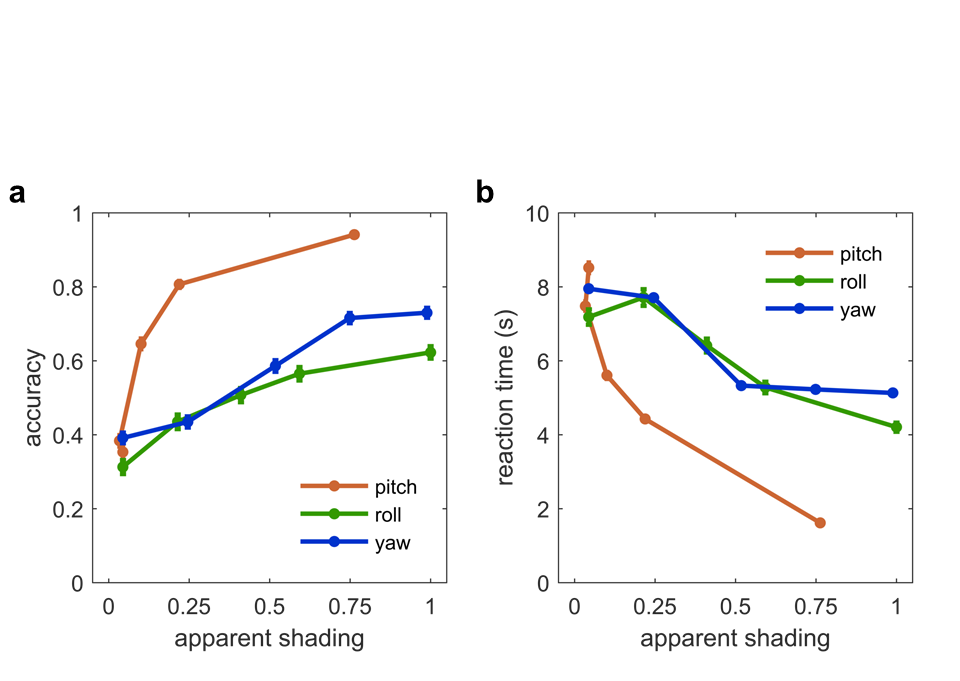


Figure S4. Relationship between search efficiency and apparent shading. (a) Observers detection accuracy against apparent shading on the target object for Experiments 1 (pitch, brown curve), Experiment 2 (roll, green curve) and Experiment 3 (yaw, blue curve). (b) Observers detection speed against apparent shading on the target object for Experiments 1, 2 and 3; colour code as in (a). As in the main text, Figure 6, the shading has been normalised with the same multiplicative factor across the three experiments such as to have a global maximum of 1. Error bars show the standard errors of the mean.
